# Solvothermal-assisted liquid-phase exfoliation of graphite in a mixed solvent of toluene and oleylamine

**DOI:** 10.1186/s11671-014-0727-9

**Published:** 2015-01-21

**Authors:** Dinh Khoi Dang, Eui Jung Kim

**Affiliations:** School of Chemical Engineering, University of Ulsan, Daehak-ro 93, Nam-gu, Ulsan 680-749 South Korea

**Keywords:** Graphene, Solvothermal-assisted exfoliation, Mixed solvent, Oleylamine, Toluene

## Abstract

We report an effective method for producing graphene sheets using solvothermal-assisted exfoliation of graphite in a mixed solvent of toluene and oleylamine. The mixed solvent of toluene and oleylamine produces higher yield of graphene than its constituents, oleylamine and toluene. The oleylamine molecules with its long chain enwrap the graphene sheets efficiently, while toluene helps the oleylamine molecules become more flexible and easily intercalate into the edge of graphite. The prepared graphene sheets have a high quality, and the concentration of graphene in the dispersion is as high as 0.128 mg mL^−1^. The high-quality graphene sheets obtained in this work make them suitable for application in many fields such as energy-storage materials and polymer composites.

## Background

Graphene, a one-atom thick, two-dimensional monolayer of sp^2^-bonded carbon, has worldwidely attracted attention of researchers due to its tremendously large surface area, good chemical stability, outstanding electrical conductivity, and high mechanical strength [1,2].

It is noticeable that, however, few existing graphene preparation methods can achieve the defect-free and large-scale production of large-size graphene. For instance, the scotch tape cleavage approach produces high-quality graphene sheets, but it suffers from a main drawback of low yield and throughput [2].

To overcome the low yield and throughput of the graphene sheets in the graphene production, chemical avenues have been developed including graphene oxide reduction [3-6], sonication-supported exfoliation [7-9], and graphite intercalation [10-12]. Among them, graphene oxide reduction is the most popular method to produce large-size graphene sheets in large quantities. However, the graphene sheets prepared by this method are highly defective, which restricts their application in electronic devices [13]. On the other hand, sonication-supported exfoliation of graphite produces high quality and nearly defect-free graphene sheets, but a major issue of low graphene concentration needs to be addressed. Since defect-free graphene is required for application in a variety of electronic devices, a cost-effective large-scale fabrication method to produce defect-free graphene needs to be developed.

Recently, the sonication-supported exfoliation of graphite in a mixed solvent environment has emerged as a potential method for producing high-quality graphene sheets [14,15]. The selection of effective solvents for graphene dispersion is based on the Hildebrand solubility parameters, the Hansen solubility parameters, and surface free energy. It is understood that the van der Waals attractive interaction between the graphene sheets needs to be weaker than the interaction between graphene and solvent.

Previous studies have reported that highly polar solvents such as 1-methyl-2-pyrrolidone (NMP) are capable of readily dispersing graphene and carbon nanotubes [8]. Similarly, aliphatic amines are also good solvents for dispersing carbon nanotubes, especially acid-treated carbon nanotubes [16-19], because they have strong interactions with the sp^2^ carbon lattice network of the carbon nanotubes. Among these aliphatic amines, oleylamine with its long chain might also be a useful solvent for graphene dispersion. A solvothermal route has been employed to synthesize graphene sheets and reduced graphene oxide at a higher temperature and pressure [13,20]. The graphite may be exfoliated more effectively under these intense conditions than under mild conditions [21].

Benefiting from co-synergistic effect of mixed solvent of oleylamine and toluene, herein, we report an effective method to enhance both the efficiency of the liquid-phase exfoliation of graphite and the quality of graphene sheets. By employing solvothermal-assisted exfoliation in a mixed solvent to control the interactions between solvent molecules and sp^2^ carbon atoms of graphite, we propose a new approach to producing graphene dispersion with high-yield and high-quality graphene sheets.

## Methods

### Materials

Expandable graphite (Grade 1721) was kindly provided by Asbury Carbon (Asbury, NJ, USA). Oleylamine, toluene, and 1-methyl-2-pyrrolidinone (NMP) were purchased from Aldrich Chemical Inc (Sigma-Aldrich, St. Louis, MO, USA). Hydrochloric acid and ethanol were purchased from DaeJung Chemicals and Metals Co. Ltd (Shiheung, Korea). All reagents and solvents were used without further purification.

### Exfoliation

Firstly, 3 g of expandable graphite was heated in a microwave for 3 min to form expanded graphite [22]. The expanded graphite was treated with hydrochloric acid by stirring for 2 days to obtain pre-intercalated graphite. Afterwards, pre-intercalated graphite (90 mg) was added to a mixed solvent (30 mL) of oleylamine and toluene with different volume ratios and was heated in an autoclave at 180°C for 24 h. After solvothermal exfoliation, the graphene solution (30 mL) was gently sonicated using a low-power sonicator bath (JEIOTECH UC-10; JEIO TECH Inc., Seoul, Korea) for 90 min to produce graphene dispersion. The resultant dispersion was centrifuged at 1,000 rpm for 30 min using a centrifuge (GYROZEN-1236MGR; GYROZEN, Daejeon, Korea). After centrifugation, the supernatant of the dispersion (15 mL) was pipetted off and then sufficiently washed with toluene/ethanol to obtain the graphene product.

### Characterization techniques

Transmission electron microscopy (TEM) images were taken on a JEM-2100 (JEOL Ltd., Seoul, Korea) with an operating voltage of 200 kV. Holey carbon films on 200 mesh copper grids (HC200-Cu; EM Systems Support Ltd., Macclesfields, UK) were employed for TEM measurements. X-ray diffraction (XRD) patterns were recorded on a Rigaku RAD-3C diffractometer (35 kV, 20 mA; Rigaku, Shibuya-ku, Japan) with Cu *Ka* radiation (λ = 1.548 Å) at a scan rate of 2°/min, in the 2*θ* angles ranging from 10° to 60°. Surface morphologies and topologies of the graphene sheets were measured by atomic force microscopy (AFM) using a multimode V (Veeco, Plainview, NY, USA) with silicon cantilevers.

UV-visible spectra were recorded on a UV-visible spectrophotometer (SPECORD 210 PLUS-223F1107; Analytik Jena AG, Jena, Germany) using a quartz cell with a 1-cm optical path. The obtained graphene product was dispersed in NMP (30 mL) for UV-visible measurements. By measuring the absorbance of graphene dispersion at 660 nm, the concentration of the exfoliated graphene dispersion was determined from the Lambert-Beer law, using *A*/*l* = *α*_660_*C*, where *A* is the absorbance, *l* [m] is the length of the optical path, *α*_660_ [=2,460 mLmg^−1^ m^−1^] is the absorption coefficient, and *C* is the concentration of graphene [8,23]. Raman spectra were taken on a confocal Raman microscope (alpha 300S; WITec, Ulm, Germany) using an incident laser light. The power and the wavelength of the laser are 6 mW and 532 nm, respectively. The aperture and the lens used for the Raman measurements are 25 μm slit and 500×, respectively.

Fourier transform infrared (FTIR) spectra were recorded on a FTIR spectrometer (KBr disk method; NICOLET 380; Thermo Fisher Scientific, Waltham, MA, USA) at wavenumbers of 400 to 4,000 cm^−1^. Thermogravimetric analysis (TGA) was conducted in nitrogen atmosphere at a heating rate of 10°C/min using a TA Hi-Res TGA 2950 thermogravimetric analyzer (TA Instruments, New Castle, DE, USA). X-ray photoelectron spectroscopy (XPS) measurement was performed on an ESCALAB 250Xi photoelectron spectrometer (Thermo Fisher Scientific, Waltham, MA, USA) with Al Kα X-ray radiation as the X-ray source for excitation.

## Results and discussion

### TEM characterization

**Figure 1:**
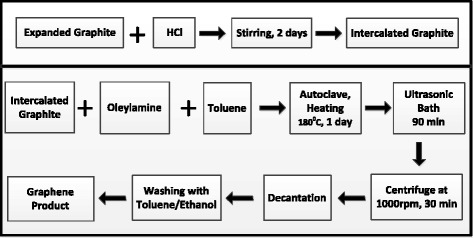
**Schematic of the process for preparation of intercalated graphite and graphene dispersion.** Top: Schematic of the process for preparation of intercalated graphite from expanded graphite. Down: Schematic of the process for preparation of graphene dispersion from intercalated graphite in a mixed solvent of toluene and oleylamine.

The exfoliation of graphite in a mixed solvent using a solvothermal process is depicted in Figure 1. In the top of Figure 1, HCl intercalates into the edge of graphite, which helps molecules intercalate into graphite in the solvothermal process. Oleylamine molecules also wrap the graphene sheets, which facilitates the exfoliation of graphite during solvothermal process, thus significantly improving the production yield of graphene.

**Figure 2:**
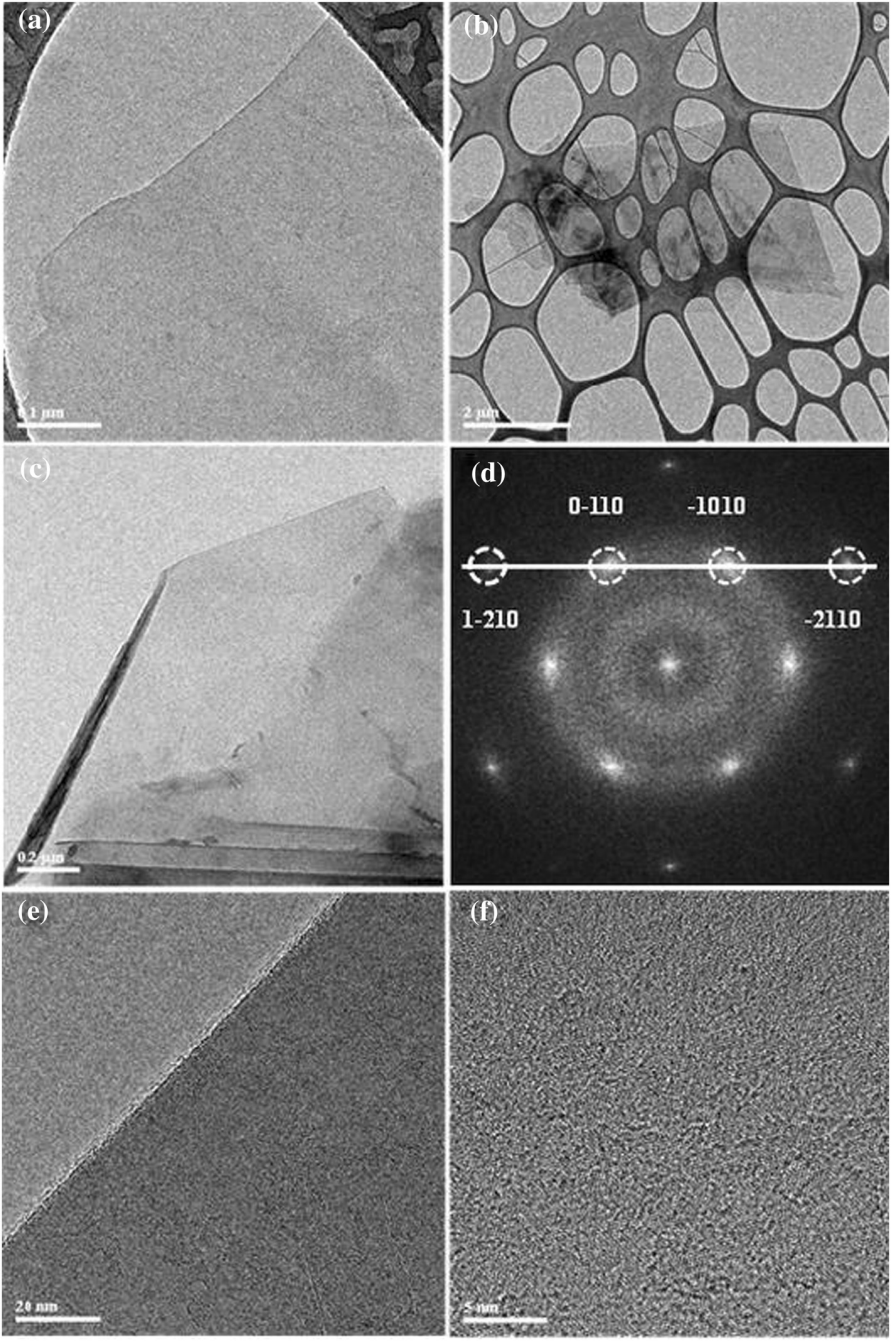
**TEM images of the prepared graphene sheets.** Samples are prepared at an optimum oleylamine/toluene ratio, which are monolayer, few-layer, and single-layer graphene sheets with a curved edge, **(a, b, c)** respectively. **(d)** Selected area electron diffraction (SAED) pattern corresponding to **(a)** with peaks labeled by the Miller-Bravais (*hkil*) indices. HRTEM images of the edge **(e)** and in-plane **(f)** of the graphene sheet taken from **(a)**.

A representative monolayer graphene sheet is shown in Figure 2a. Similarly, Figure 2b,c shows a few-layer graphene sheet and single-layer graphene sheet with a curved edge, respectively. Typical selected area electron diffraction (SAED) pattern (Figure 2d) corresponding to the Figure 2a, with the zone axis of [0001] shows that the prepared graphene is single crystalline, which is similar to that of mechanically cleaved graphene, demonstrating that the as-prepared graphene sheets have good crystallinity [24]. The SAED pattern shows a sixfold symmetry typical of graphene. The high-resolution TEM (HRTEM) image of the edge of a single-layer graphene sheet is shown in Figure 2e. The HRTEM image of the prepared graphene sheets indicates that the graphene product has a high quality and crystallinity, which is clearly demonstrated by a perfect crystal lattice in HRTEM image (Figure 2f).

### AFM characterization

**Figure 3:**
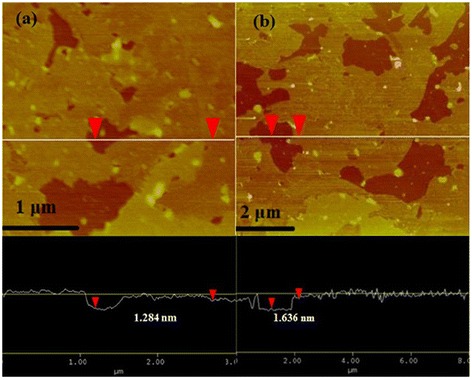
**Two typical AFM images of graphene sheets with a height profile taken along the straight line (a, b).** The sample was prepared by drop-casting dilute graphene dispersion onto a silicon wafer.

The thickness of the prepared graphene sheets was measured by AFM. Figure 3a,b shows two typical tapping-mode AFM images of few-layer graphene sheets prepared at an optimum oleylamine-to-toluene ratio. Height profiles taken along the straight line show that the thickness of the graphene sheet is around 1.20 to 1.60 nm. This indicates that the graphene sample is a few-layer sheet (no more than five layers) [1,25], which is consistent with the TEM results.

### Concentration of graphene product

**Figure 4:**
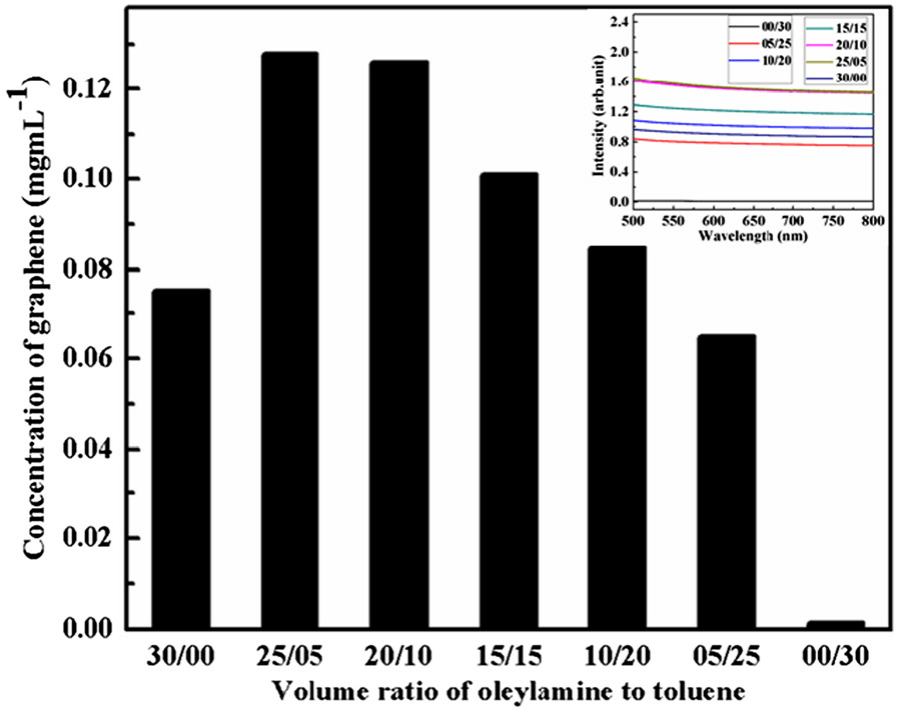
**Concentration of graphene sheets at various volume ratios of oleylamine to toluene.** The inset is the UV spectra of graphene sheets at various volume ratios of oleylamine to toluene.

The concentration of the graphene product at various volume ratios of toluene to oleylamine is shown in Figure 4. It can be seen from Figure 4 that the concentration of graphene reaches the highest value of 0.128 mg mL^−1^ when the volume ratio of oleylamine/toluene is 5, which is much higher than the reported value (0.01 mg mL^−1^) for a solvent of NMP [8]. This value of graphene concentration for the mixed solvent of toluene and oleylamine is higher than that for pure solvents, oleylamine (0.075 mg mL^−1^), and toluene (1.14 × 10^−3^ mg mL^−1^).

Due to the strong ionic interactions between strong acid and oleylamine, the oleylamine molecules readily intercalate into the graphite layers at high temperature and pressure in the solvothermal process. Moreover, with its long chain, oleylamine can prevent the re-aggregation of the graphene sheets effectively, making the graphene suspension more stable.

For successful exfoliation of graphite, it is necessary to overcome the van der Waals attractions between adjacent graphite layers. One method to reduce the strength of the van der Waals attractions is liquid immersion. By matching the refractive indices of the material and the solvents, the potential energy between adjacent layers which is given by the dispersive London interactions may approach 0. Based on this criterion, solvents with a surface tension of 40 to 50 mJm^−2^ were suggested for the dispersion of carbon nanotubes and graphene [26,27]. The surface tension of mixed oleylamine/toluene solvents is measured as 27 to 31 mJm^−2^, which is lower that the suggested surface tension for the exfoliation of graphite. In view of surface tension, oleylamine and toluene do not seem to be good solvents for the exfoliation of graphite. However, under solvothermal conditions, the oleylamine molecules readily intercalate into the graphite layers, which facilitates the exfoliation of graphite. The addition of toluene into oleylamine makes the oleylamine molecule more flexible, thus increasing the effectiveness of the exfoliation process. Because toluene alone can poorly disperse graphene, the graphene concentration decreases as the toluene content in the mixed solvent increases, i.e., the volume ratio of oleylamine to toluene decreases.

### Raman characterization

**Figure 5:**
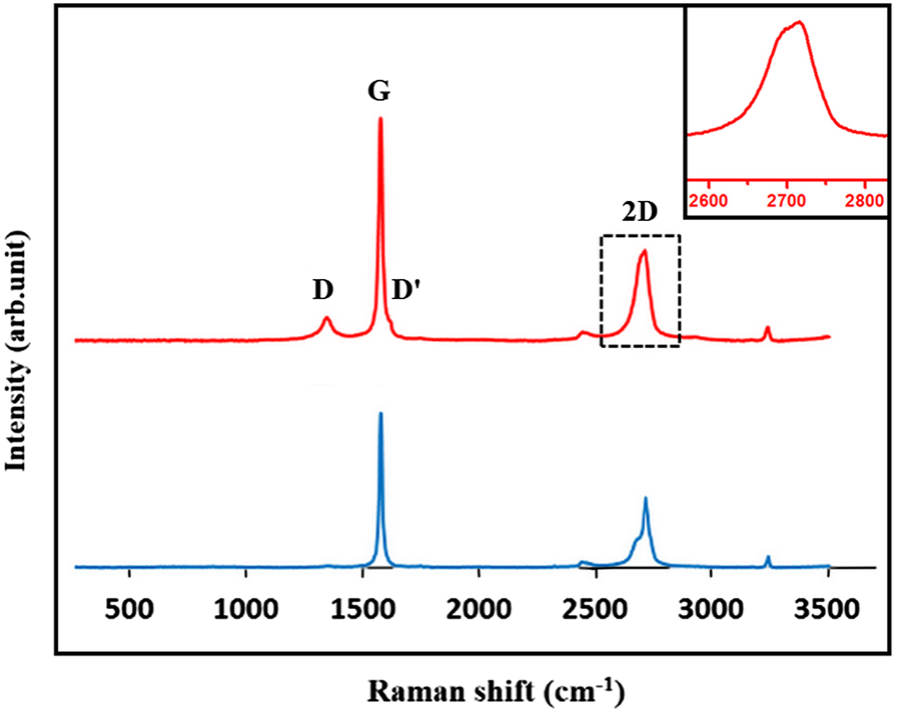
**Raman spectra of graphite (down) and graphene product (top).** The inset is a magnification of the black dashed rectangle area.

Raman spectra of the prepared graphene sheets are shown in Figure 5. It can be seen from Figure 5 that the D-band appears at approximately 1,350 cm^−1^, the G-band at approximately 1,580 cm^−1^, and the 2D-band at approximately 2,700 cm^−1^ in both Raman spectra of graphite and graphene product. The D′-band at approximately 1,620 cm^−1^ is observed in the Raman spectrum of graphene product [28]. Low D-band intensities indicate that the prepared graphene sheets contain few defects. The intensity ratio of D to D′ band (*I*_D_/*I*_D′_) is found to be 1.14. A low value of *I*_D_/*I*_D′_ indicates that the defects are on-site defects, which describe out-of-plane atoms bonded to carbon atoms (namely, sp^3^ hybridization) [29]. Moreover, the intensity ratio of D′ and G (*I*_D′_/*I*_G_) is 0.09, indicating that the defects are mostly on sp^3^ sites, because in the same defect concentration, *I*_D′_/*I*_G_ is higher for vacancies than sp^3^ sites [30]. The intensity ratio of D to G band (I_D_/I_G_) is found to be 0.11. This ratio is much lower than that of chemically or thermally reduced graphene oxide (approximately 1.2 to 1.5) and graphene prepared by other exfoliation methods, further demonstrating that our graphene product contains a low amount of defects [30-32]. An enlarged view of the 2D-band in the inset of Figure 5 is similar to the shape of bilayer graphene sheet in previous work [24]. This indicates that our graphene products are few-layer graphene sheets (less than five layers) because the Raman spectrum of graphene sheets with a thickness of more than five layers are hardly distinguished from that of bulk graphite [9,24,25,28].

### FTIR characterization

**Figure 6:**
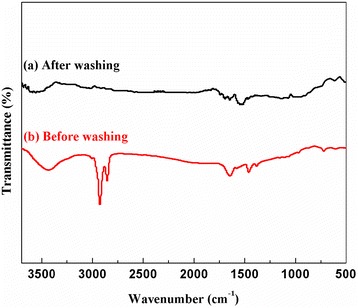
**FTIR spectra of the prepared graphene sheets (b) before and (a) after washing with toluene/ethanol.**

Figure 6 shows the FTIR spectra of the prepared graphene before and after washing with toluene/ethanol. Three main peaks appear in both spectra. A peak at approximately 3,445 cm^−1^ is attributed to O-H stretching vibration of adsorbed water molecules and structural OH groups, and a peak at approximately 1,647 cm^−1^ is ascribed to C=C bending vibrations. Peaks at approximately 1,525 cm^−1^ reflect the skeletal vibration of graphene [33]. It is observed from FTIR spectra of the product before washing that besides those three peaks, there are also two peaks at around 2,920 cm^−1^ and 2,850 cm^−1^ which are attributed to stretching of methyl group in oleylamine [34-37] and toluene, indicating that toluene and oleylamine remain in the sample before washing. Also, there are peaks at around 1,500 cm^−1^ which may be attributed to NH_2_ bending mode [35] indicating that the final graphene product contains a small amount of oleylamine after washing.

### XRD characterization

**Figure 7:**
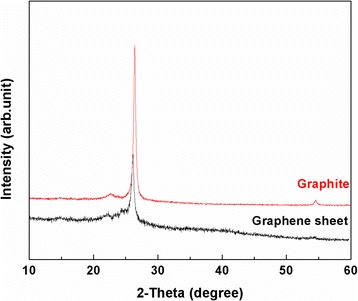
**XRD patterns of graphite and the prepared graphene sheets.**

Figure 7 shows the XRD patterns of graphite and the prepared graphene. It can be seen from XRD patterns that the diffraction peak of the prepared graphene is slightly shifted to a lower diffraction angle compared with the pattern of graphite, indicating an increase in the interplanar spacing determined from Bragg's law (2dsinθ = nλ). Although the interlayer spacing of graphite is increased to a limited extent, the increased interlayer spacing is favorable for the organic molecules (toluene and oleylamine) to intercalate into the lattice of graphite, thus facilitating the exfoliation of graphite for the production of the graphene sheets. The peak intensity of the prepared graphene is decreased compared with that of graphite due to the delamination of pristine graphite into a few-layer graphene sheets [23,38]. The XRD results in Figure 7 indicate that oleylamine intercalates into interplanar spaces of graphite.

### TGA characterization

**Figure 8:**
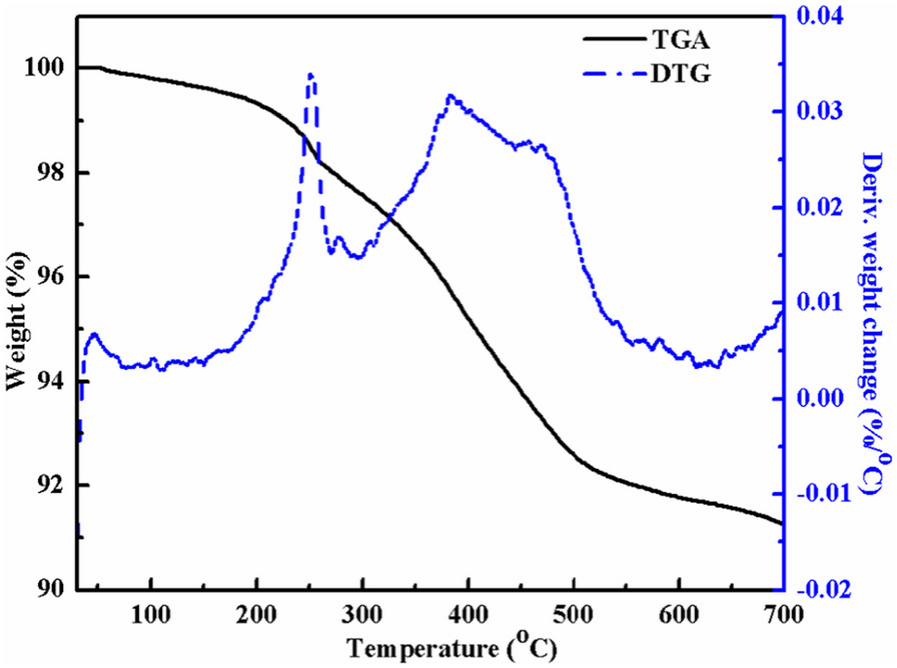
**Thermogravimetric curves of the prepared graphene sheets.**

TGA results (Figure 8) reveal that the prepared graphene loses weight by approximately 8% at 450°C~550°C, which is comparable to our previous report [23]. The initial mass loss around 250°C may be due to the loss of epoxy groups (C-O-C) and amine group (NH_2_) and the second loss around 400°C to 500°C may be attributed to the loss of carboxyl (C=O) and carboxylic groups (O-C=O) [39,40]. The interaction between graphene and solvent should result in the existence of amine group in the final graphene product. The C-O-C, C=O, and O-C=O groups may come from the adsorption of oxygen onto the surface of the graphene sheets from the air during the synthesis process.

### XPS characterization

**Figure 9:**
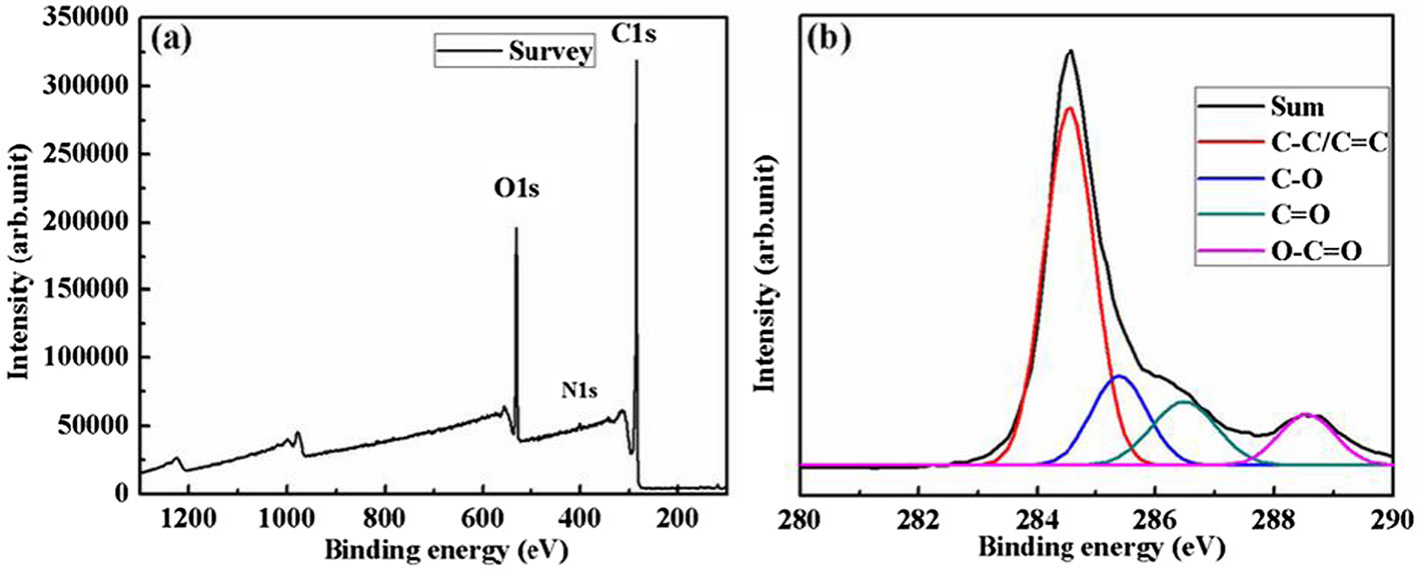
**XPS survey spectrum (a) and C1s XPS spectrum (b) of the prepared graphene sheets.**

XPS analysis was performed to monitor changes of the structural and chemical composition of the sample during the preparation process. Figure 9a shows the XPS survey spectrum of the prepared graphene containing a large amount of C (82.39%) and a small amount of O (16.84%). This is comparable to thermally reduced graphene oxide at 1,100°C in vacuum [41] and the graphene obtained by liquid phase exfoliation of graphite in surfactant/water solutions [9]. There also exists an extremely small amount of N (0.76%), which indicates that the graphene product contains a very small amount of oleylamine after washing. Figure 9b shows the C1s XPS spectrum of the prepared graphene sheets. The C1s peak can be decomposed into four component peaks with a binding energy of 284.54, 285.39, 286.48, and 288.55 eV, which are assigned to C-C/C=C, C-O, C=O, and O-C=O bonds, respectively [39]. The intensity of the C-C/C=C peak is very large compared to that of the C-O, C=O, and O-C=O peaks indicating that the graphene product contains a very small amount of oxygen on the surface.

## Conclusions

In conclusion, we have demonstrated an effective and efficient approach to preparing high-concentration graphene dispersion by solvothermal exfoliation of graphite in a mixed solvent of toluene and oleylamine. The prepared graphene dispersion contains mono or few-layer graphene sheets with a low amount of defects. The graphene concentration in the dispersion is as high as 0.128 mg mL^−1^ at a volume ratio of oleylamine and toluene of 5. This result is comparable to or higher than previously reported values. An oleylamine molecule with its long chain can intercalate into the edge of graphite during solvothermal process and also wrap the graphene sheets. The addition of toluene, a poor solvent for graphene dispersion, into oleylamine helps oleylamine become more flexible and thus significantly improves the production yield of graphene. High-quality graphene sheets prepared in this work may find their application in many areas from energy-storage materials to polymer composites.

## References

[1] Novoselov KS, Geim AK, Morozov SV, Jiang D, Zhang Y, Dubonos SV (2004). Electric field effect in atomically thin carbon films. Science..

[2] Novoselov KS, Jiang D, Schedin F, Booth TJ, Khotkevich VV, Morozov SV (2005). Two-dimensional atomic crystals. Proc Natl Acad Sci U S A..

[3] Li D, Muller MB, Gilje S, Kaner RB, Wallace GG (2008). Processable aqueous dispersions of graphene nanosheets. Nat Nano..

[4] Luo Z, Lu Y, Somers LA, Johnson ATC (2009). High yield preparation of macroscopic graphene oxide membranes. J Am Chem Soc..

[5] Tung VC, Allen MJ, Yang Y, Kaner RB (2009). High-throughput solution processing of large-scale graphene. Nat Nano..

[6] Zhou X, Liu Z (2010). A scalable, solution-phase processing route to graphene oxide and graphene ultralarge sheets. Chem Commun..

[7] Tang YB, Lee CS, Chen ZH, Yuan GD, Kang ZH, Luo LB (2009). High-quality graphenes via a facile quenching method for field-effect transistors. Nano Lett..

[8] Hernandez Y, Nicolosi V, Lotya M, Blighe FM, Sun Z, De S (2008). High-yield production of graphene by liquid-phase exfoliation of graphite. Nat Nano..

[9] Lotya M, Hernandez Y, King PJ, Smith RJ, Nicolosi V, Karlsson LS (2009). Liquid phase production of graphene by exfoliation of graphite in surfactant/water solutions. J Am Chem Soc..

[10] Vallés C, Drummond C, Saadaoui H, Furtado CA, He M, Roubeau O (2008). Solutions of negatively charged graphene sheets and ribbons. J Am Chem Soc..

[11] Li X, Zhang G, Bai X, Sun X, Wang X, Wang E (2008). Highly conducting graphene sheets and Langmuir-Blodgett films. Nat Nano..

[12] Hao R, Qian W, Zhang L, Hou Y (2008). Aqueous dispersions of TCNQ-anion-stabilized graphene sheets. Chem Commun..

[13] Wang H, Robinson JT, Li X, Dai H (2009). Solvothermal reduction of chemically exfoliated graphene sheets. J Am Chem Soc..

[14] Yi M, Shen Z, Ma S, Zhang X (2012). A mixed-solvent strategy for facile and green preparation of graphene by liquid-phase exfoliation of graphite. J Nanoparticle Res.

[15] Oyer AJ, Carrillo JMY, Hire CC, Schniepp HC, Asandei AD, Dobrynin AV (2012). Stabilization of graphene sheets by a structured benzene/hexafluorobenzene mixed solvent. J Am Chem Soc..

[16] Basiuk EV, Basiuk VA, Bañuelos JG, Saniger-Blesa JM, Pokrovskiy VA, Gromovoy TY (2002). Interaction of oxidized single-walled carbon nanotubes with vaporous aliphatic amines. J Phys Chem B..

[17] LeMieux MC, Roberts M, Barman S, Jin YW, Kim JM, Bao Z (2008). Self-sorted, aligned nanotube networks for thin-film transistors. Science..

[18] Ju SY, Utz M, Papadimitrakopoulos F (2009). Enrichment mechanism of semiconducting single-walled carbon nanotubes by surfactant amines. J Am Chem Soc..

[19] Kong J, Dai H (2001). Full and modulated chemical gating of individual carbon nanotubes by organic amine compounds. J Phys Chem B..

[20] Choucair M, Thordarson P, Stride JA (2009). Gram-scale production of graphene based on solvothermal synthesis and sonication. Nat Nanotechnol..

[21] Qian W, Hao R, Hou Y, Tian Y, Shen C, Gao H (2009). Solvothermal-assisted exfoliation process to produce graphene with high yield and high quality. Nano Res..

[22] Bang GS, So HM, Lee MJ, Ahn CW (2012). Preparation of graphene with few defects using expanded graphite and rose bengal. J Mater Chem..

[23] Xu J, Dang DK, Tran VT, Liu X, Chung JS, Hur SH (2014). Liquid-phase exfoliation of graphene in organic solvents with addition of naphthalene. J Colloid Interface Sci..

[24] Ferrari AC, Meyer JC, Scardaci V, Casiraghi C, Lazzeri M, Mauri F (2006). Raman spectrum of graphene and graphene layers. Phys Rev Lett..

[25] Ni ZH, Wang HM, Kasim J, Fan HM, Yu T, Wu YH (2007). Graphene thickness determination using reflection and contrast spectroscopy. Nano Lett..

[26] Coleman JN (2009). Liquid-phase exfoliation of nanotubes and graphene. Adv Funct Mater..

[27] Cai M, Thorpe D, Adamson DH, Schniepp HC (2012). Methods of graphite exfoliation. J Mater Chem..

[28] Malard LM, Pimenta MA, Dresselhaus G, Dresselhaus MS (2009). Raman spectroscopy in graphene. Phys Rep..

[29] Eckmann A, Felten A, Mishchenko A, Britnell L, Krupke R, Novoselov KS (2012). Probing the nature of defects in graphene by Raman spectroscopy. Nano Lett..

[30] Eckmann A, Felten A, Verzhbitskiy I, Davey R, Casiraghi C (2013). Raman study on defective graphene: effect of the excitation energy, type, and amount of defects. Phys Rev B..

[31] Mattevi C, Eda G, Agnoli S, Miller S, Mkhoyan KA, Celik O (2009). Evolution of electrical, chemical, and structural properties of transparent and conducting chemically derived graphene thin films. Adv Funct Mater..

[32] López V, Sundaram RS, Gómez-Navarro C, Olea D, Burghard M, Gómez-Herrero J (2009). Chemical vapor deposition repair of graphene oxide: a route to highly-conductive graphene monolayers. Adv Mater..

[33] Tang Z, Zhuang J, Wang X (2010). Exfoliation of graphene from graphite and their self-assembly at the oil-water interface. Langmuir..

[34] Zhang JL, Srivastava RS, Misra RDK (2007). Core-shell magnetite nanoparticles surface encapsulated with smart stimuli-responsive polymer: synthesis, characterization, and LCST of viable drug-targeting delivery system. Langmuir..

[35] Xu Z, Shen C, Hou Y, Gao H, Sun S (2009). Oleylamine as both reducing agent and stabilizer in a facile synthesis of magnetite nanoparticles. Chem Mater..

[36] Shukla N, Liu C, Jones PM, Weller D (2003). FTIR study of surfactant bonding to FePt nanoparticles. J Magn Magn Mater..

[37] Mourdikoudis S, Liz-Marzán LM (2013). Oleylamine in nanoparticle synthesis. Chem Mater..

[38] Liu WW, Wang JN (2011). Direct exfoliation of graphene in organic solvents with addition of NaOH. Chem Commun..

[39] Luan VH, Tien HN, Hur SH (2015). Fabrication of 3D structured ZnO nanorod/reduced graphene oxide hydrogels and their use for photo-enhanced organic dye removal. J Colloid Interface Sci..

[40] Luan VH, Tien HN, Hoa LT, Hien NTM, Oh ES, Chung J (2013). Synthesis of a highly conductive and large surface area graphene oxide hydrogel and its use in a supercapacitor. J Mater Chemistry A..

[41] Becerril HA, Mao J, Liu Z, Stoltenberg RM, Bao Z, Chen Y (2008). Evaluation of solution-processed reduced graphene oxide films as transparent conductors. ACS Nano..

